# Utilization of affordable nanocomposites with outstanding antimicrobial activity in waterborne coatings

**DOI:** 10.1038/s41598-025-05819-y

**Published:** 2025-06-25

**Authors:** Walaa M. Abd El-Gawad

**Affiliations:** Polymers and pigments Dept, National Research Centre, Dokki, Cairo Egypt

**Keywords:** Silica fume industrial waste, Nanocomposites, Nano-ZnO, Nano-CuO, Antimicrobial activity, Waterborne coatings, Chemistry, Materials science, Nanoscience and technology

## Abstract

This study aimed to explore the development of eco-friendly antimicrobial coatings by combining antimicrobial nanocomposites with waterborne resins. Novel nanocomposites, such as nano-ZnO/silica fume and nano-CuO/silica fume, were synthesized using the solution combustion method, along with pure nano-ZnO and nano-CuO. The nanocomposites consist of a thin layer of nanometal oxide on silica fume, aiming to enhance antimicrobial activity. These nanocomposites were incorporated into acrylic waterborne resin at two concentrations (0.4 and 0.8 wt%) to provide cost-effective alternatives to imported and expensive antimicrobial agents. Antimicrobial effectiveness was evaluated against *Staphylococcus aureus*,* Micrococcus luteus*,* and Candida albicans* using disc diffusion and shake flask methods. Besides, the mechanical and physical properties of the coatings were compared to the properties of a commercial coating. The findings showed that the commercial coating offered inhibition zones ranging from 16 to 21 mm. While the disc containing 0.8% nano-ZnO/silica fume offered the greatest antimicrobial activity, with inhibitory zones ranging from 17 to 26.6 mm. Additionally, the results demonstrated that discs containing nano-ZnO were better than discs containing nano-CuO. The mechanical properties indicated that the hardness of coatings with either nano-ZnO or nano-CuO is similar to the commercial coatings in group I. However, coatings with nano-ZnO/silica fume and nano-CuO/silica fume exhibited slightly higher hardness. In group II, higher ratios of nano-ZnO, nano-CuO, and their silica fume composites significantly increase hardness compared to the commercial coatings, attributed to the formation of a more compact film. Moreover, the results showed that coatings with a high ratio of pigments (0.8%) adhered better than those with 0.4% of pigments.

## Introduction

The increasing awareness about healthcare and severe damages caused by pathogenic microorganisms has forced the scientists to examine the mechanism of biological damage and its protection^1^. Among the different protection approaches, the use of antimicrobial coatings has been a key component in inhibiting the growth as well as accumulation of harmful microorganisms on the surfaces^2^.

Moreover, to improve the benefits of antimicrobial coatings towards the environment, the choice of an appropriate solvent in the formulation of the coatings is of utmost importance^3^. Using organic solvents in coatings leads to the emission of volatile organic compounds (VOCs), which could cause severe health and environmental problems. VOCs inhalation can lead to several kinds of health problems, including headaches, asthma attacks, skin and eye irritation, as well as harm to the liver, kidneys, or nervous system^4^. Since water is abundant, affordable, and, most importantly, non-toxic, it is seen to be the perfect substitute for organic solvents. The main advantages of waterborne coatings over solvent-based alternatives are such as low to zero VOCs content, non-flammable, and low odor^5–7^. The implementation of strict environmental regulations that reduce the emission of VOCs from coatings is expected to enhance the demand of the market of waterborne coatings^3,6^. In spite of their increasing popularity, the typical shortcomings of latex coatings, such as water sensitivity, low solvent resistance and flash rust formation often limit their application^8^. Therefore, the development of waterborne antimicrobial coatings with good film performance has gained spark due to the negative environmental effects of solvent-based coatings, as demonstrated by a significant amount of research articles on this topic^9^.

Recently, antimicrobial coatings are developed by incorporating bactericidal agents, such as antibiotics, nanoparticles, etc^10–12^. Metal-based nanoparticles have garnered significant interest over recent decades due to their exceptional physical and chemical properties, including antimicrobial activity. Various metal-based nanoparticles, such as CuO, TiO_2_, and ZnO, have been extensively studied and successfully utilized as antibacterial agents. Among these, ZnO nanoparticles are widely used in hygiene coatings and are generally considered safe for humans, as affirmed by the Food and Drug Administration (FDA). Notably, nano-ZnO exhibits strong antimicrobial properties without the need for light activation, in contrast to TiO_2_, which requires illumination. Additionally, nanostructured ZnO possesses other important characteristics, including high chemical stability, photocatalytic activity, and significant ultraviolet absorption. As a result, ZnO nanoparticles find applications in a broad range of fields, such as catalysts, pigments, optical materials, cosmetics, UV absorbers, and various industrial additives^8,13,14^. Similarly, nano-CuO displays unique physicochemical properties due to the quantum size effect and high specific surface area, enhancing its biological and chemical activity. Another key feature of nano-CuO is its ability to target different types of bacterial structures^15^.

However, the synthesis of these nanomaterials involves high costs, complicated processes, a high ratio of heavy metals, etc. Therefore, the development of new antimicrobial nanomaterials with improved antibacterial activity, ease of production, and low cost is in demand to limit the expenditure of the precious nanomaterials^16^.

Therefore, the present work focused on synthesizing novel antimicrobial nanocomposites (e.g., nano-ZnO/silica fume and nano-CuO/silica fume) by using core-shell technology to reduce the cost of nanomaterials and the consumption of expensive nano-oxides by depositing a very thin layer of either nano-ZnO or nano-CuO on the surface of silica fume industrial waste. Additionally, this method has the potential to convert hazardous industrial silica fume waste into functional materials with antibacterial properties. These nanocomposites have a low ratio of heavy metals because they were made of a thin layer of nano-ZnO or nano-CuO that was deposited on the surface of silica fume industrial waste. Following the synthesis step, the prepared pure nano-ZnO, nano-CuO, and nanocomposites were integrated into acrylic waterborne coatings with two ratios (e.g., 0.4 and 0.8 wt%) to replace the expensive antimicrobial agent.

## Experimental part

### Materials

Silica fume is an industrial waste recovered from the exhausted gases of ferrosilicon factory in Aswan, Egypt. Zinc nitrate and copper nitrate were purchased from LOBA Chemi, India with purity 99% and 99.9%, respectively. Urea was obtained from Rasayan Laboratories in India. The commercial antibacterial coating called “Magico Anticoo” was bought from Pachin Co., Egypt. Perimal E 822 K was supplied by DOW, U.S. Propylene glycol with purity ≥ 99.5% (GC) was obtained from Sigma-Aldrich, U.S.A. All the used extenders and solvents were supplied by local and international companies with normal chemical grades.

### Synthesis of the nanopigments and nanocomposites using combustion solution method

The analytical pure grade of all compounds was employed without further purification. A brand-new combustion technique was used to create ZnO and CuO nanoparticles. In a typical process, urea and 0.3 M [zinc nitrate or copper nitrate] and urea with molar ratio (1 metal nitrate :1 urea) were mixed. The combinations were then burned for ½ h at 200 °C. The entire mixture was then annealed for two hours at 550 °C. The combustion reaction resulted into white fluffy ZnO powder and black fluffy CuO powder^17,18^.

**Fig. 1:**
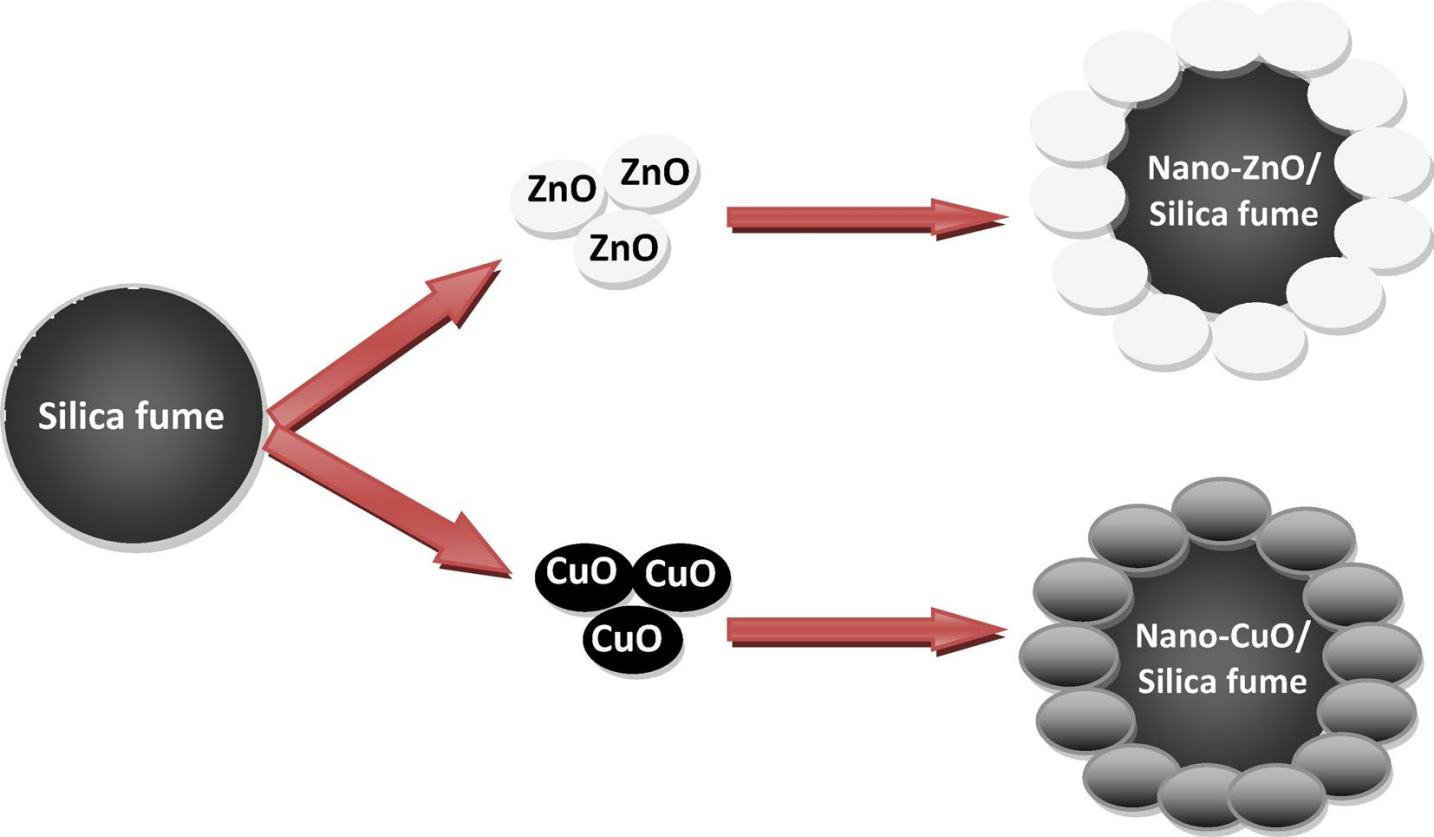
Nano-ZnO/silica fume and nano-CuO/silica fume.

To prepare nano-ZnO/silica fume and nano-CuO/silica fume, the same steps were done but in the presence of 90 g of silica fume. The weight of chemicals and silica fume was adjusted stoichiometrically to precipitate a thin layer of ZnO or CuO nanoparticles with almost 10% silica fume, which comprises almost 90% of the whole compounds. The following equation is an example of a combustion reaction. A schematic diagram of the synthesized nanocomposites is given in Fig. 1.

In this preparation method the metal nitrate hydrate is act as an oxidizer, and urea (CH_4_N_2_O) as a fuel. This reaction which is thermal explosion can be performed in two stages. The first mode is occurred where the entire volume of the reactive mixture is preheated uniformly at 200 °C. This stage is followed by a relatively long constant temperature rang where free and a portion of the bound water evaporates. The next stage is characterized by a higher rate (ignition temperature at 550 °C) to remove all organic species and have the metal oxide. The following equation is an example for combustion reaction.

$$\:{Z}{n}{\left({N}{{O}}_{3}\right)}_{2}.6{{H}}_{2}{O}+{C}{{H}}_{4}{{N}}_{2}{O}\mathop{\rightarrow}^{\Delta}\:\:{Z}{n}{O}+{C}{{O}}_{2}+8{{H}}_{2}{O}+{{2N}}_{2}+{{O}}_{2}$$

### The instrumentation part

Transmission electron microscopy (TEM) and scanning electron microscopy (SEM)/energy-dispersive X-ray analysis (EDX) techniques using micro-analyzer electron probes (JEOL JX 1230 and JEOL JX 2840) in Japan, respectively, were used to determine the shapes and sizes of the prepared nanocomposites. The metal oxide concentration in the prepared nanocomposites was determined using an Axios sequential WDX-ray fluorescence (XRF) spectrometer, PANalytical 2005. X-ray powder diffraction patterns (XRD) were obtained at room temperature using a Philip’s diffractometer (Model PW1390), employing Ni-filtered Cu Kα radiation (λ = 1.5404 A°). The diffraction angle, 2θ, was scanned at a rate of 2°/min. Perkin Elmer thermogravimetric analyzer TGA7 technique, USA, was used. FT-IR spectra of the prepared compounds were obtained with a JASCO FTIR-4100 E FT-IR spectrometer (Japan) operating in absorption mode in the wave number range of 4,000–400 cm^−1^ by the prepared compounds mixing with KBr (potassium bromide) discs.

### Paint formulations

Herein, eight formulations of antimicrobial glossy coatings were performed as shown in Table 1. The antimicrobial agent in the commercial one was replaced by the prepared pigments (e.g., nano-ZnO, nano-ZnO/silica fume, nano-CuO, and nano-CuO/silica fume) in two ratios (0.4 and 0.8 wt%). First, the weighted nanopigments or nanocomposites were mixed with a definite volume of water using ultrasonic for 0.5 h to enhance the dispersion of the nanomaterials in the formulations. After that, the other ingredients were added to every dispersed solution with the acrylic resin and mixed using a ball mill for 2 hoursto prepare the coatings. Then, the coatings were filtered to make sure there were no coagulated particles. Finally, the formulations were painted on a plastic substrate using a film applicator with a thickness of 120 μm to determine their antimicrobial activity. The newly prepared antimicrobial coatings were compared to a commercial antimicrobial coating^19^. The formulation recipe is shown in Table 1.

**Table 1:** Paint formulations.

Ingredients (gm)/paints	Commercial coating (Standard)	Group I				Group II			
		1	2	3	4	5	6	7	8
Water	18.25	18.25	18.25	18.25	18.25	18.25	18.25	18.25	18.25
Tetra pot. Pyro phosphate	0.1	0.1	0.1	0.1	0.1	0.1	0.1	0.1	0.1
Orotan 731 A	0.6	0.6	0.6	0.6	0.6	0.6	0.6	0.6	0.6
Liquid non ionic alkyd aryl	0.3	0.3	0.3	0.3	0.3	0.3	0.3	0.3	0.3
Anti-bacteria	0.4	–	–	–	–	–	–	–	–
Defoamer	0.6	0.6	0.6	0.6	0.6	0.6	0.6	0.6	0.6
Propylene glycol	2	2	2	2	2	2	2	2	2
Extender	14	14	14	14	14	13.6	13.6	13.6	13.6
P-H965	13	13	13	13	13	13	13	13	13
Hydroxyl meth.	0.3	0.3	0.3	0.3	0.3	0.3	0.3	0.3	0.3
Ropaque O P 96	6	6	6	6	6	6	6	6	6
Ucarlatex D- 153	20	20	20	20	20	20	20	20	20
Perimal E 822 K	20	20	20	20	20	20	20	20	20
2-amino-2-methil-1propanol	0.1	0.1	0.1	0.1	0.1	0.1	0.1	0.1	0.1
Acrysol TT 935	0.124	0.124	0.124	0.124	0.124	0.124	0.124	0.124	0.124
Zinc Omadme Zoe Disp.	2	2	2	2	2	2	2	2	2
P-D612	0.2	0.2	0.2	0.2	0.2	0.2	0.2	0.2	0.2
Texanol	2	2	2	2	2	2	2	2	2
Tintaid	0.01	0.01	0.01	0.01	0.01	0.01	0.01	0.01	0.01
Tinting paste	0.016	0.016	0.016	0.016	0.016	0.016	0.016	0.016	0.016
Nano-ZnO	–	0.4	–	–	–	0.8	–	–	–
nano-ZnO/silica fume	–	–	0.4	–	–	–	0.8	–	–
Nano-CuO	–	–	–	0.4	–	–	–	0.8	–
nano-CuO/silica fume	–	–	–	–	0.4	–	–	–	0.8

### Antimicrobial measurements method

The antimicrobial assessments of the produced coatings containing nano-ZnO, nano-ZnO/silica fume, nano-CuO, or nano-CuO/silica fume were carried out against the three strains, such as *Staphylococcus aureus*,* Micrococcus luteus*, which are bacterial strains, and *Candida albicans*, which is a pathogenic fungus, using the disc diffusion method in nutrient agar plates. The inoculation plates were then incubated at 37 °C for 24 h, after which the zones of inhibition were measured in millimetres^20^.

The shake flask method was used to determine the actual antimicrobial percentage for the negative samples with zero inhibition zone in the disc diffusion method to calculate the antimicrobial activities expressed throughout the (%) reduction of bacterial count by calculating colony-forming units (CFU) of these tested strains after treatment with the tested samples compared to the number of microorganism cells surviving in the control flask after a 24 h incubation period and at 37 °C for bacteria and pathogenic yeast^21–23^.

The difference between both techniques is that the disc diffusion method gives inhibition zones for samples that can offer 100% inhibition. While CFU is done for the samples that do not have clear inhibition zones, this technique gives the actual percentage of antimicrobial activity, which is less than 100%.

### Physical properties measurements

A LovibondTintometer RT 100 Color tintometer was used to spectrally analyze the color of the coatings via the CIELab method. The L* value in this method represents the color lightness on a scale of 0 to 100, where 100 signifies a superior reflecting diffuser and zero, the lowest L* value, denotes a black or dark color. a* and b*, which are at right angles to one another and cross in the middle, stand in for the other axes. It is predicated on the rule that a color cannot be both red and green, or blue and yellow. Positive values of a* depict red, whereas negative values are represented by green. Positive values of b* correspond to yellow, whereas negative values correspond to blue. Furthermore, according to ASTM D523, the gloss test, a crucial aspect of surface appearance, was performed. In accordance with ASTM D1200, the viscosity was assessed using a KerbsStormer viscometer.

### Mechanical properties determination

The mechanical characteristics (e.g. hardness [ASTM D4366], impact resistance [ASTM D2794], ductility [ASTM E643] and pull-off strength [ASTM D4541]) were determined to figure out the elasticity, strength and flexibility and of the coated films containing the prepared pigments.

## Results and discussion

### Characterization of the nanopigments and nanocomposites

#### XRD

**Fig. 2:**
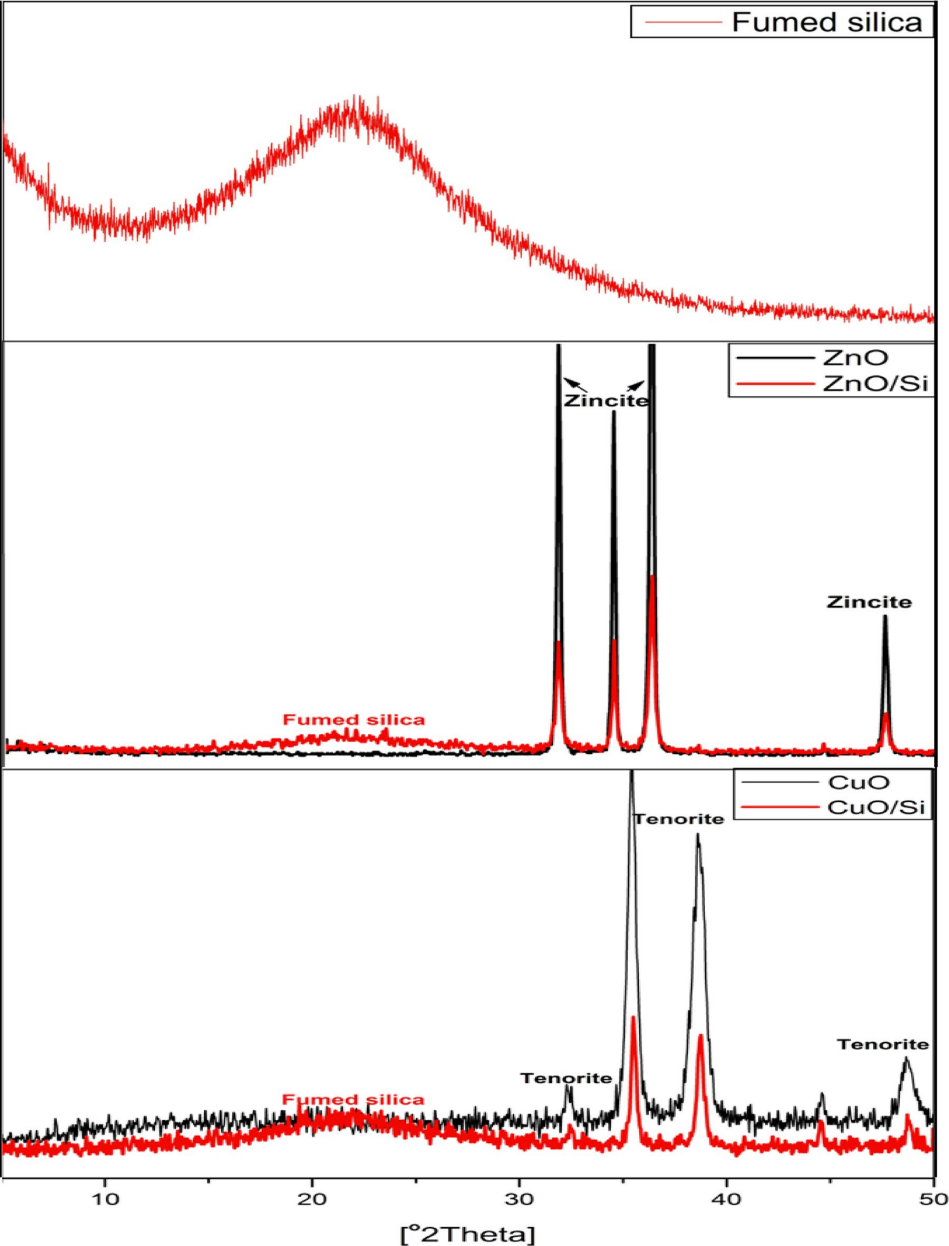
XRD of silica fume, nano-ZnO, nano-ZnO/silica fume, nano-CuO and nano-CuO/silica fume.

The XRD pattern of synthesized pigments is shown in Fig. 2. The XRD pattern of silica fume revealed that its bands are wide, confirming silica’s amorphous nature. In the instance of nano-ZnO, the narrow and sharp diffraction peaks indicate that nano-ZnO has a desirable crystallinity, and they signify the impacts of controlled conditions on the growth of crystals. The nano-ZnO pattern exhibits noticeable peaks at 2θ values of 31.64°, 34.43°, 36.29°, and 47.75°, and they match the standard JCPDS card no. 01–79-0206^24^. Additionally, no characteristic peaks other than ZnO appear, which in turn specifies its high purity.In the case of nano-ZnO/silica fume, additional broad bands of silica fume appeared in the range of 2θ values of 10°−30°.On the other hand, patterns of nano-CuO showed the presence of various characteristic peaks at 2θ values of 32.5°, 35.5°, 38.7°,48.6° which can be indexed on the basis of orthorhombic copper (II) oxide (JCPDS file No. 05–661)^25^, and also broad bands of silica fume were noticed in the case of nano-CuO/silica fume.

#### SEM-TEM

**Fig. 3:**
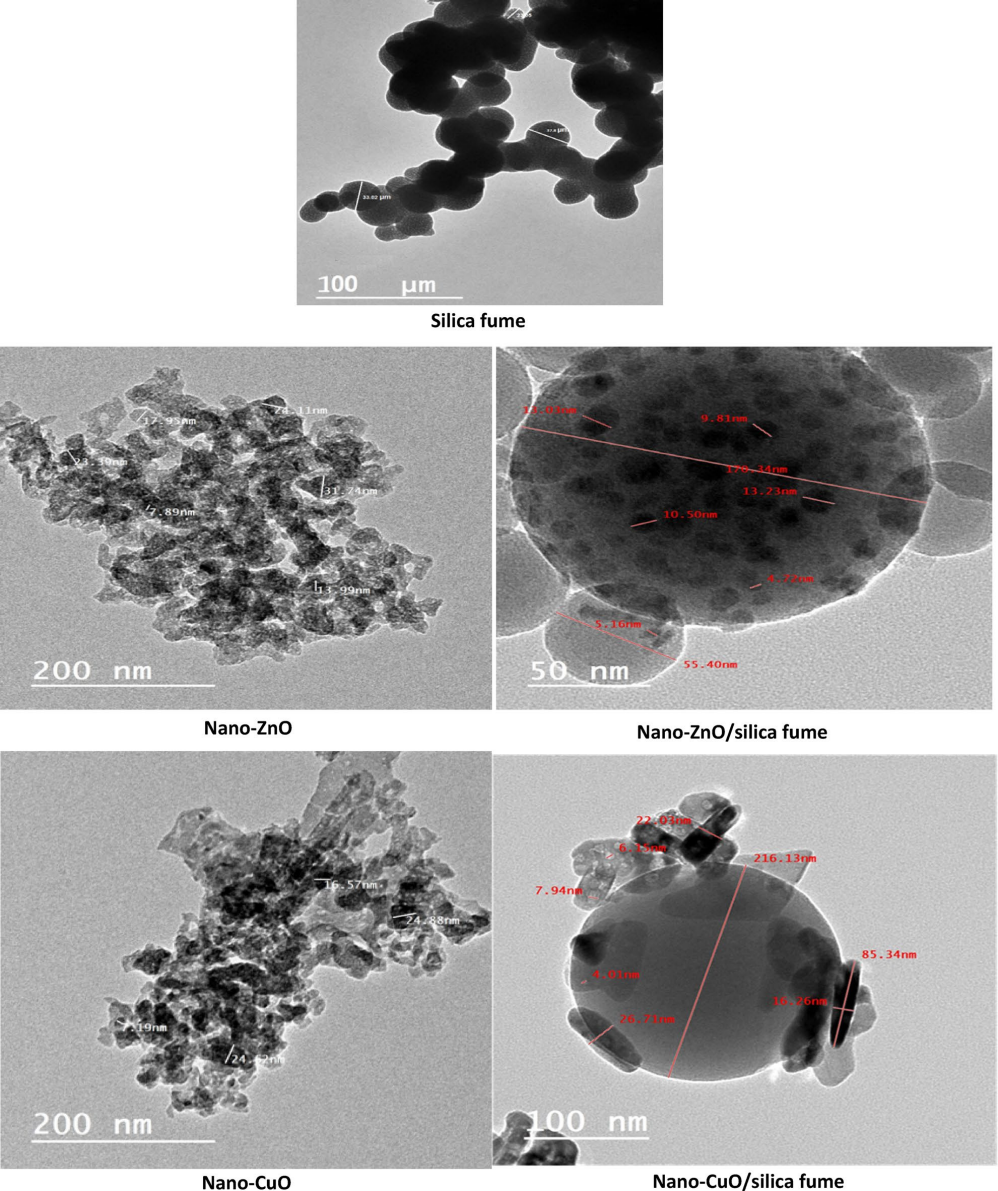
TEM of silica fume, nano-ZnO, nano-ZnO/silica fume, nano-CuO and nano-CuO/silica fume.

**Fig. 4:**
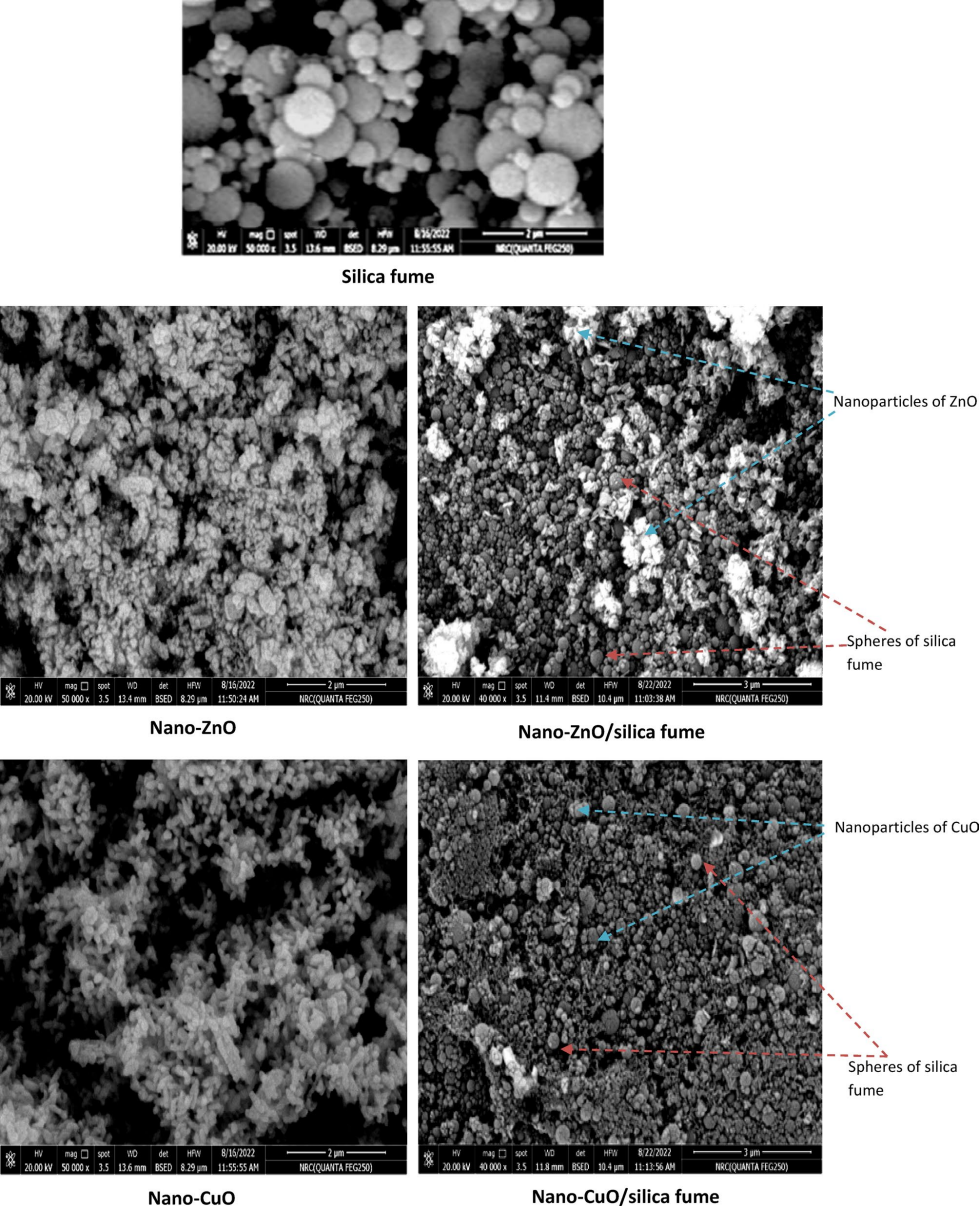
SEM of silica fume, nano-ZnO, nano-ZnO/silica fume, nano-CuO and nano-CuO/silica fume.

Figures 3 and 4 present the morphology and particle size of the prepared pigments. The featured photo of SEM and TEM showed that silica fume particles have a spherical shape on the micron scale. Moreover, SEM photos indicated that particles of nano-ZnO and nano-CuO are plates, while those of nanocomposites showed that the surface of micronized spherical silica fume was covered with the platelet particles of the different oxides on a nanoscale. On the other hand, TEM was used to investigate the particle shape and size.The featured photos demonstrate that nano-ZnO and nano-CuO particles have platelet shapes on a nanoscale. In the case of nanocomposites, the appearance of both shapes (e.g., the spherical shape of silica fume and the platelet shape of the prepared nano-oxides) confirm the synthesis of nanocomposites. In this case the spherical particles of silica fume were completely covered with oxides, and the nano oxide particles were methodically overlapping, connecting, and enclosing the spherical particles of silica fume.

#### EDX

**Fig. 5:**
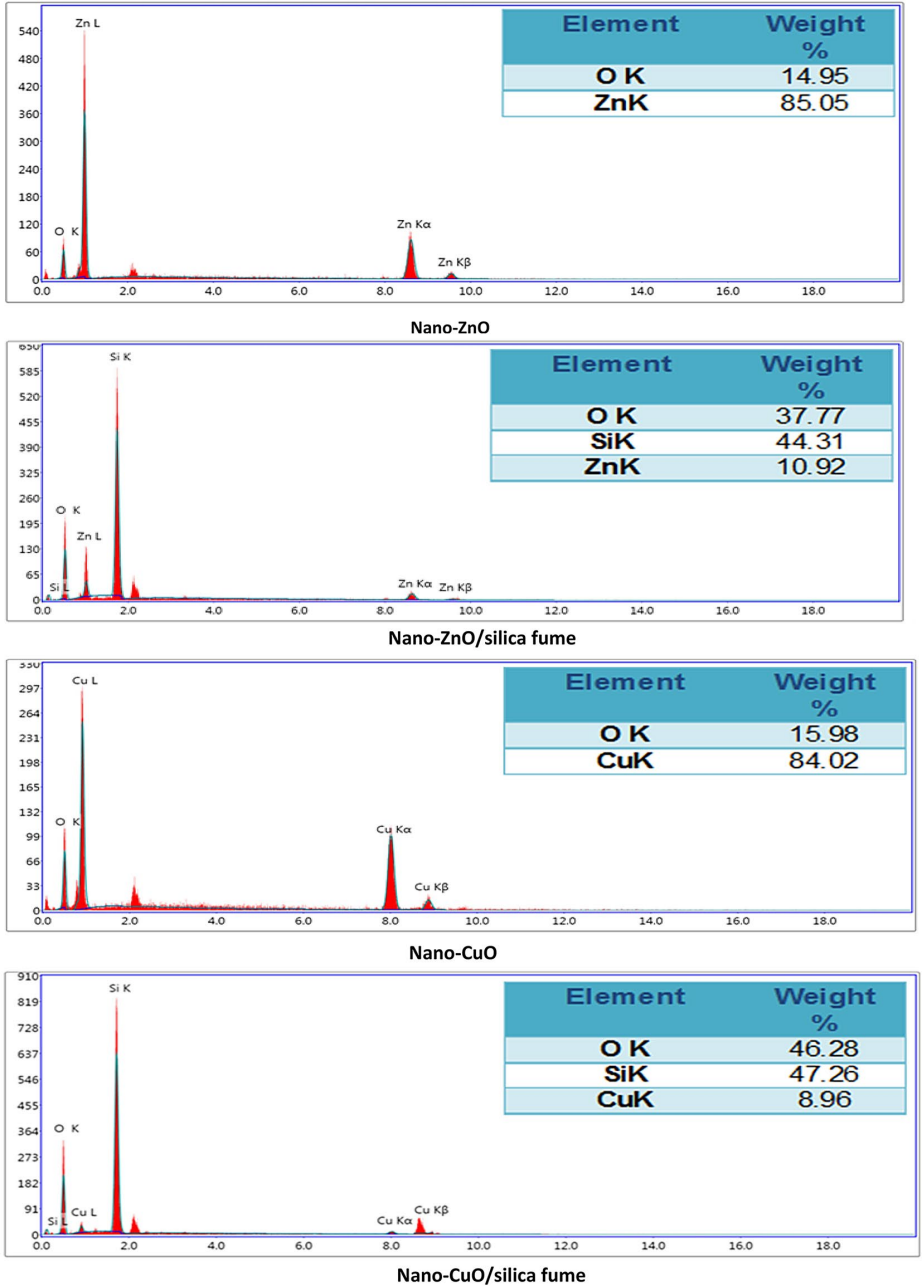
EDX of silica fume, nano ZnO, nano-ZnO/silica fume, nano-CuO and nano-CuO/silica fume.

As depicted in Fig. 5, the EDX micrographs of nano-ZnO and nano-CuO reveal their purity. Both Zn and O are present in nano-ZnO with weight percentages of 85.05% and 14.98%, respectively, proving that the prepared ZnO is essentially free from impurities. Also, only Cu and O peaks appeared in nano-CuO. In the case of the prepared ZnO/silica fume and nano-CuO/silica fume, an additional peak of Si appeared with a high ratio, which confirms that a thin layer of the mixed nano-oxides were successfully precipitated on the surface of silica fume, which is the predominant constituent.

#### XRF

The XRF results in Table 2 showed the concentration of the oxides in the prepared pigments. XRF analysis of silica fume showed that silica fume is composed of SiO_2_ with 96.75% traces of other oxides. While XRF of nano-ZnO and nano-CuO pigments showed their preparation with ratios of 96.6 and 96.8, respectively, besides traces of other oxides. In the case of ZnO/silica fume and nano-CuO/silica fume nanocomposites, SiO_2_ appeared with a high ratio in addition to a low percentage of ZnO or CuO, which is in agreement with EDX results and proves that a thin layer of the mixed nano-oxides was deposited on the surface of silica fume.

**Table 2:** XRF analysis of silica fume, nano-ZnO, nano-ZnO/silica fume, nano-CuO and nano-CuO/silica fume.

Main constituents (wt%)	SiO_2_	ZnO	CuO	Al_2_O_3_	Fe_2_O_3_	Cl		CaO	MgO	SO_3_	K_2_O	TiO_2_	*P*_2_O_5_	LOI
Silica fume	96.75	–	0.02	0.39	1.04	0.31	0.32		0.43	0.03	0.53	0.11	0.07	
Nano-ZnO	0.01	96.69	–	0.131	0.86	0.213	0.345		0.178	0.681	0.21	–	0.02	0.76
Nano-ZnO/silica fume	83.4	11.4	–	0.319	1.18	0.13	0.698		0.483	0.172	0.668	0.04	0.03	1.45
Nano-CuO	0.83	0.0662	96.89	0.371	0.0682	0.049	0.769		0.34	0.121	0.12	–	0.0483	0.32
Nano-CuO/silica fume	83.3	0.29	10.9	0.342	1.05	0.084	0.694		0.589	0.341	0.689	0.18	0.06	1.44

#### TGA

**Fig. 6:**
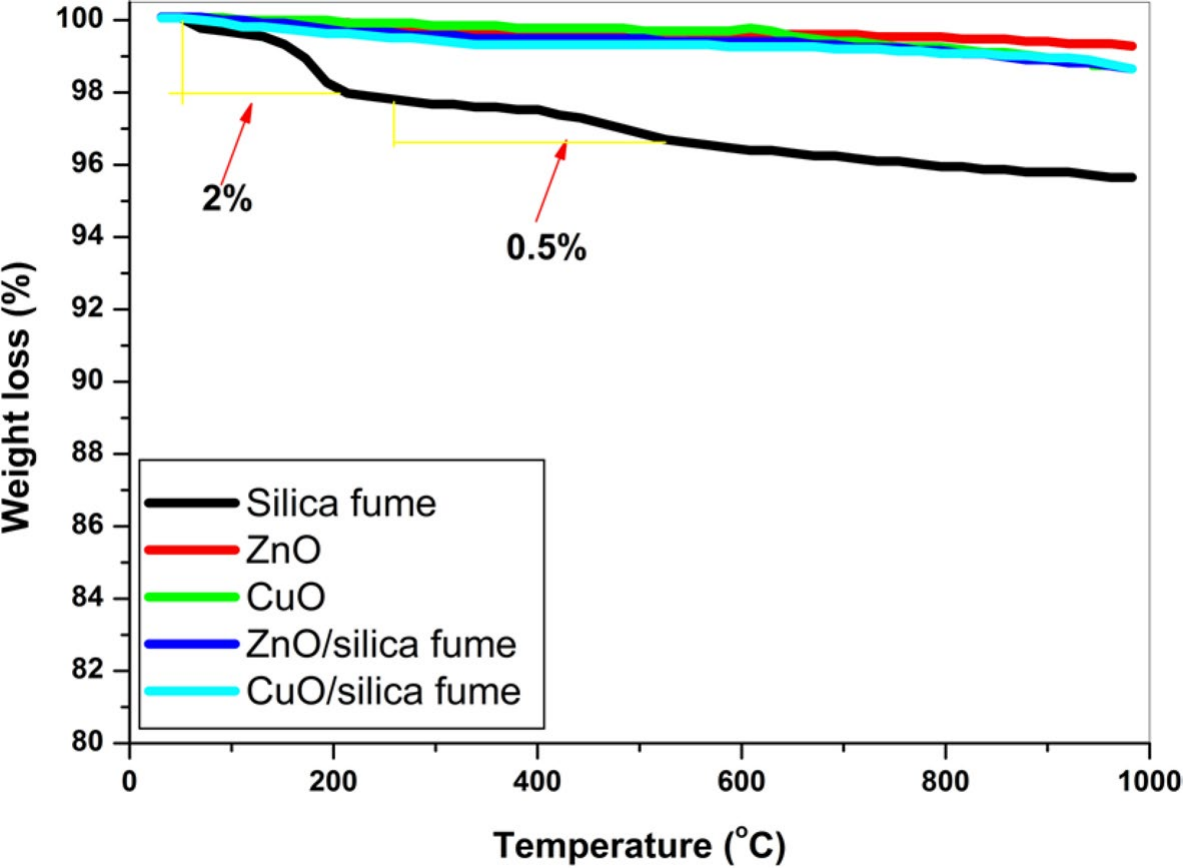
TGA of silica fume, nano-ZnO, nano-ZnO/silica fume, nano-CuO and nano-CuO/silica fume.

Figure 6 exhibits the TGA weight loss curves of silica fume nano-ZnO, nano-ZnO/silica fume, nano-CuO, and nano-CuO/silica fume. The weight loss of silica fume was almost 2.5%, while the prepared pigments have insignificant weight loss. These results indicate that the prepared nanocomposites are thermally stable, and the deposition of nano-ZnO and nano-CuO on the surface of silica fume enhances the thermal stability.

#### FTIR

**Fig. 7:**
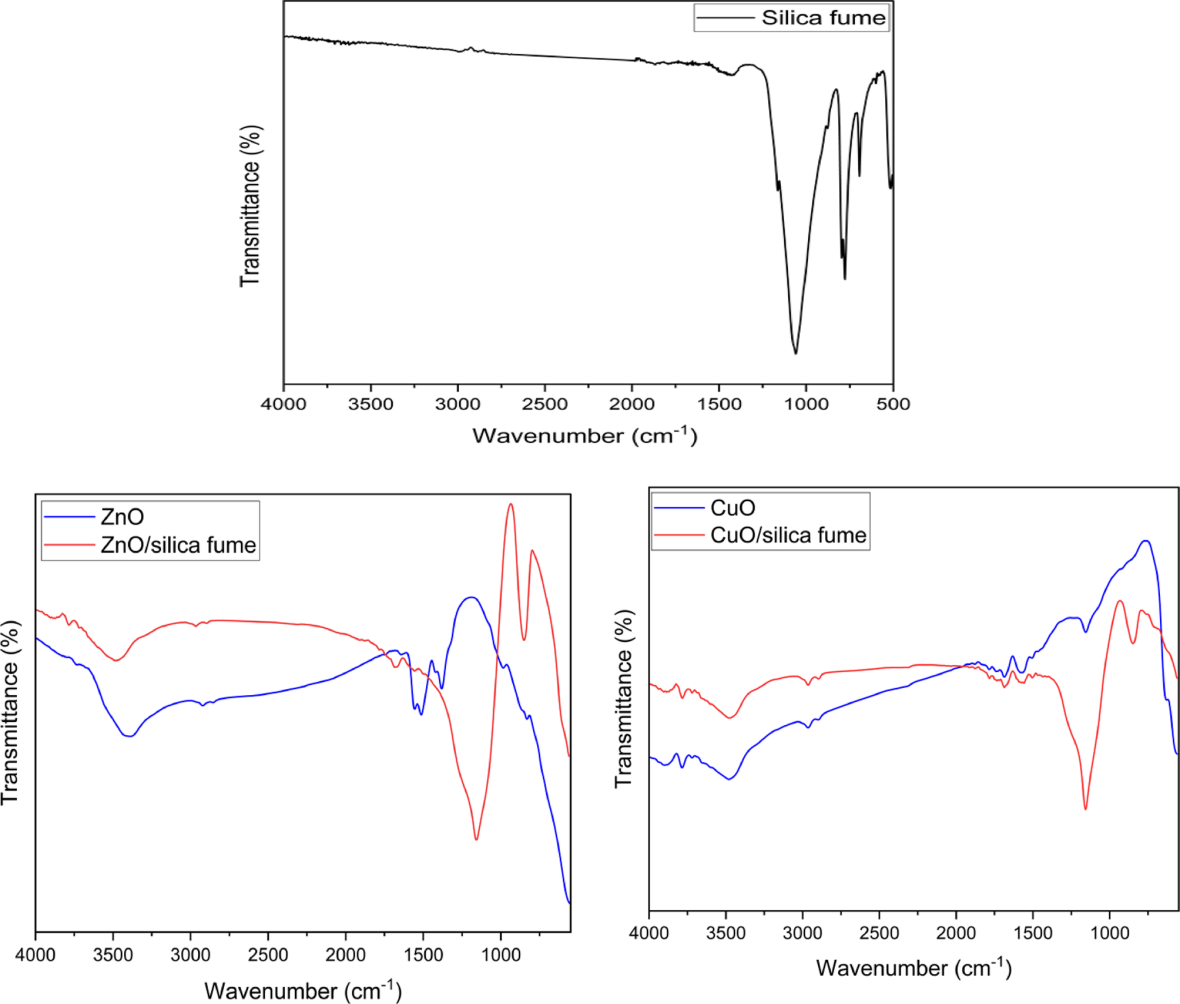
FTIR of silica fume, nano-ZnO, nano-ZnO/silica fume, nano-CuO and nano-CuO/silica fume.

In Fig. 7, the FT-IR spectrum of silica fume, nano-ZnO, nano-CuO, nano-ZnO/silica fume and nano-CuO/silica fume is reported. The chart of silica fume shows that the band in the region between 1250 and 1100 cm^−1^ can be attributed to the Si–O–Si bonds (symmetric and asymmetric stretching). The bands at about 470 and 800 cm^−1^ are absorption bands of Si–O vibrations and the band near 1625 cm^−1^ is attributed to the bending vibration of the adsorbed water molecule^26^.

In the case of nano-ZnO and nano-CuO, alcohol in-plane bend or vibration appeared at 1118.1 cm⁻¹, 1355.5 cm⁻¹, and 1383.7 cm⁻¹. Besides, the peak at 1595.8 cm^−1^ is due to the vibration mode of the alkyl group. Both peaks at 2925.8 cm^−1^ and 3437.8 cm^−1^ are related to the hydroxyl compounds stretching vibration^27^. In nano-ZnO, ZnO stretching vibrations appeared at 876 cm^−1^^28^ and the chart of nano-CuO confirms that nano-CuO has an absorption peak at 584 cm^−1^^29^. Meanwhile, in nano-ZnO/silica fume and nano-ZnO/silica fume, the characteristic peaks of both silica and metal oxides appeared together with a slight shift due to the comination between both of them.

### Evaluation of antimicrobial activity

**Fig. 8:**
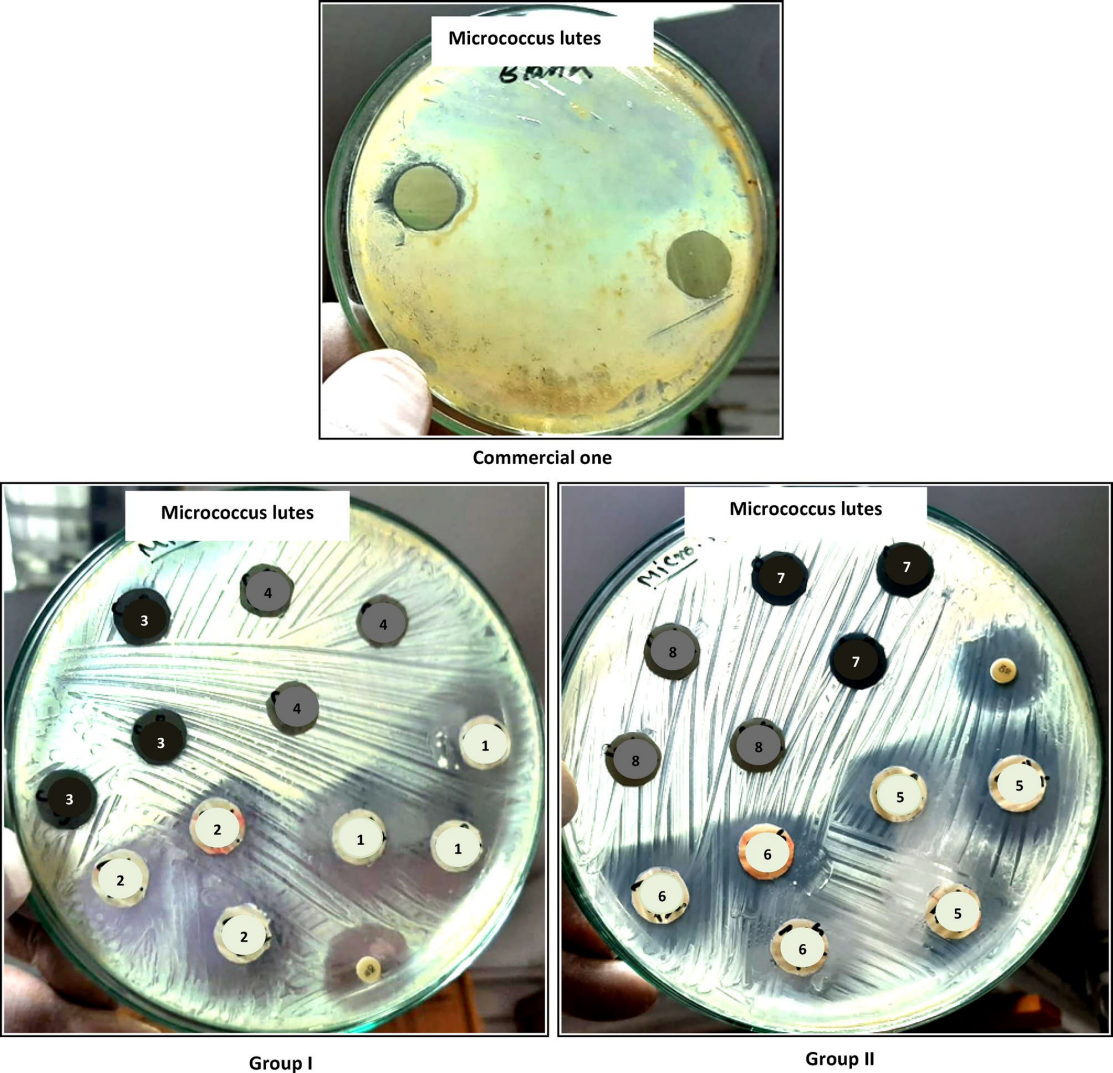
Inhibition zone of discs containing the prepared nanopigments and nanocomposites against *Micrococcus lutes.*

**Fig. 9:**
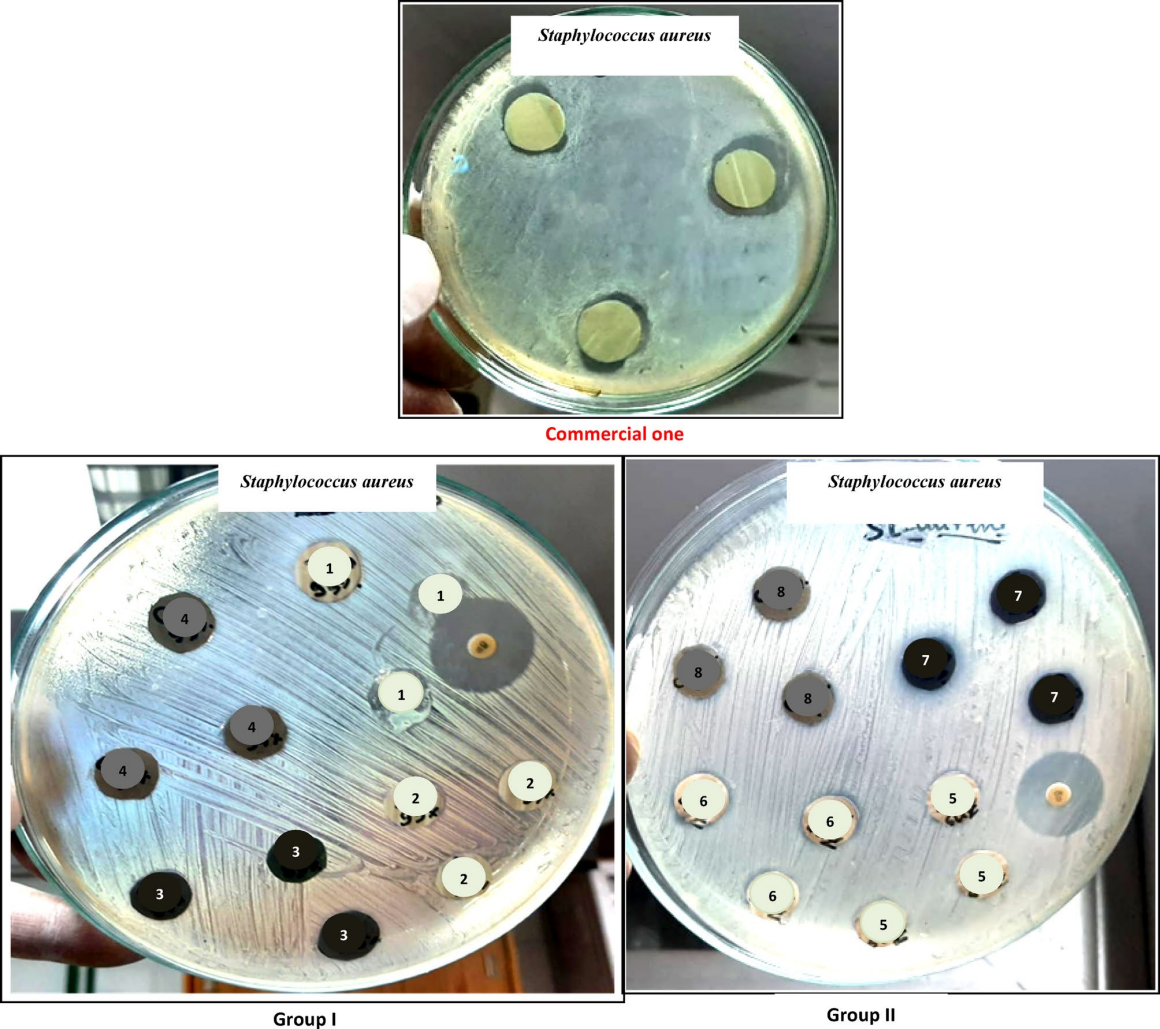
Inhibition zone of discs containing the prepared nanopigments and nanocomposites against *Staphylococcus aureus*

**Fig. 10:**
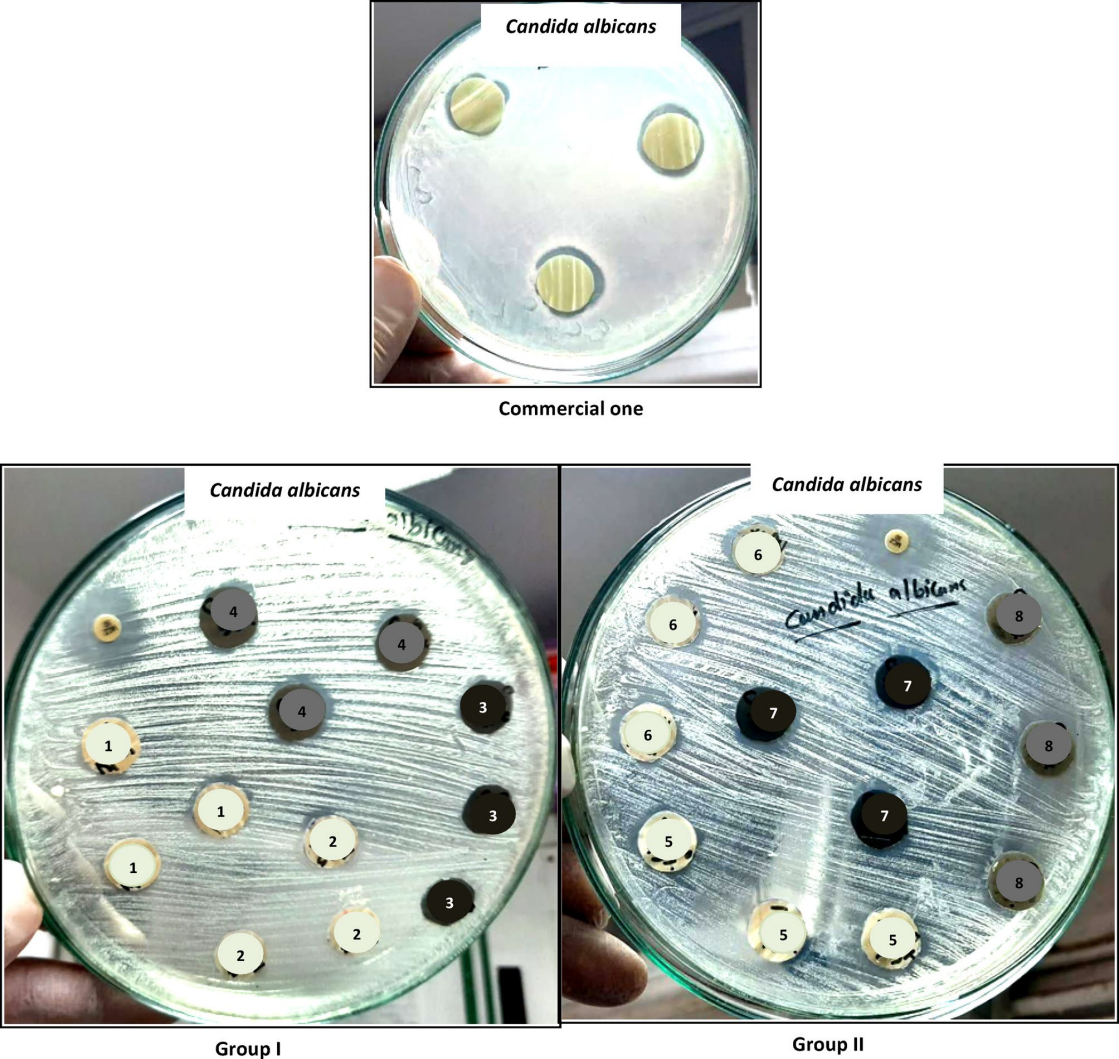
Inhibition zone of discs containing the prepared nanopigments and nanocomposites against *Candida albicans*

The antimicrobial activity of the commercial and the prepared coatings was evaluated against *Micrococcus luteus*, *Staphylococcus aureus* and *Candida albicans*. Table 3 and Figs. 8, 9, 10 show that the commercial coating offers inhibition zones of 16, 21 and 19 mm against *Micrococcus luteus*, *Staphylococcus aureus* and *Candida albicans*, respectively. Moreover, the discs containing 0.8% nano-ZnO/silica fume have good antimicrobial activity with inhibitory zones of 26.6 mm against *Micrococcus luteus*, 17 mm against *Staphylococcus aureus* and 20.6 mm against *Candida albicans*. These results demonstrate that 0.8% nano-ZnO/silica fume can be referred as broad-spectrum antimicrobial agent since it is effective against both bacteria and fungus. Besides, the inhibition zone of discs containing 0.8% nano-ZnO was 18.6–23.6 mm.

**Table 3:** Inhibition zone diameter (millimeter) of the discs containing nanopigments and nanocomposites.

Test bacteria	Samples	Commercial one	Group I				Group II				
			Nano-ZnO (1)	ZnO/Si (2)	Nano-CuO (3)	CuO/Si (4)	Nano-ZnO (5)	ZnO/Si (6)		Nano-CuO (7)	CuO/Si (8)
1	***Micrococcus luteus***	16	26	22.6	Nil	Nil	23.6		26.6	Nil	Nil
2	***Staphylococcus aureus***	21	Nil	Nil	Nil	Nil	Nil		17	19	Nil
3	***Candida albicans***	19	16.6	18.3	Nil	15	18.6		20.6	18.6	18

According to the kind of microbes, in the case of *Micrococcus luteus* and *Candida albicans*, 0.8% nano-ZnO/silica fume was better than or equal to the commercial coating, respectively. Furthermore, the results show that 0.4% nano-CuO and 0.4% nano-CuO/silica fume did not offer any antimicrobial activity against all microorganisms except that containing 0.4% nano-CuO/silica fume and gave an inhibition zone (15 mm) against *Candida albicans*. Moreover, 0.8% nano-CuO exhibited good antimicrobial activity against *Candida albicans* and *Staphylococcus aureus*, ranging from 18 to 19 mm, respectively. From the obtained findings, it is clear that discs containing nano-ZnO were better than discs containing nano-CuO. Moreover, discs containing 0.8% nano-ZnO/silica fume were the best, and their antimicrobial activity is better than the commercial one. 0.8% nano-ZnO/silica fume is constructed from approximately 10% ZnO on 90% industrial waste (silica fume), so its results are satisfactory, and it can be considered a good antimicrobial and cost-effective pigment.

CFU results in Table 4 demonstrate that coatings containing nano-ZnO and nano-ZnO/silica fume give a higher antimicrobial percentage, ranging from 88.51 to 94.95%, than those containing nano-CuO and nano-CuO/silica fume. Besides, the antimicrobial percentage increased in Group II, which means that activity increased by increasing the concentration of the prepared pigments. The manner of action of the synthesized pigments results in a disruption in the microbial cell wall, which is a peptidoglycan molecule known as murein. The nanocomposites may pass into the cells of microbes and cease several cascade instances by preventing nucleic acid generation, which subsequently results in inhibition of protein production and leads to the suppression of other metabolic actions^20^. The findings obtained reveal that the nanocomposites produced have the ability to combat microbes by altering the structure of the membrane of a cell and annihilating the growth process due to damaging the membrane structure.

**Table 4:** (%) CFU reduction of microbial strains after incubation applying the nanopigments and nanocomposites using shake flask method.

Test bacteria	Samples	Commercial one	Group I				Group II				
			Nano-ZnO (1)	ZnO/Si (2)	Nano-CuO (3)	CuO/Si (4)	Nano-ZnO (5)	ZnO/Si (6)		Nano-CuO (7)	CuO/Si (8)
1	***Micrococcus luteus***	–	–	–	55.23	55.08	–		–	75.43	75.90
2	***Staphylococcus aureus***	–	90.13	88.51	43.42	41.96	–		94.95	–	54.28
3	***Candida albicans***	–	–	–	50.81	–	–		–	–	–

**Fig. 11:**
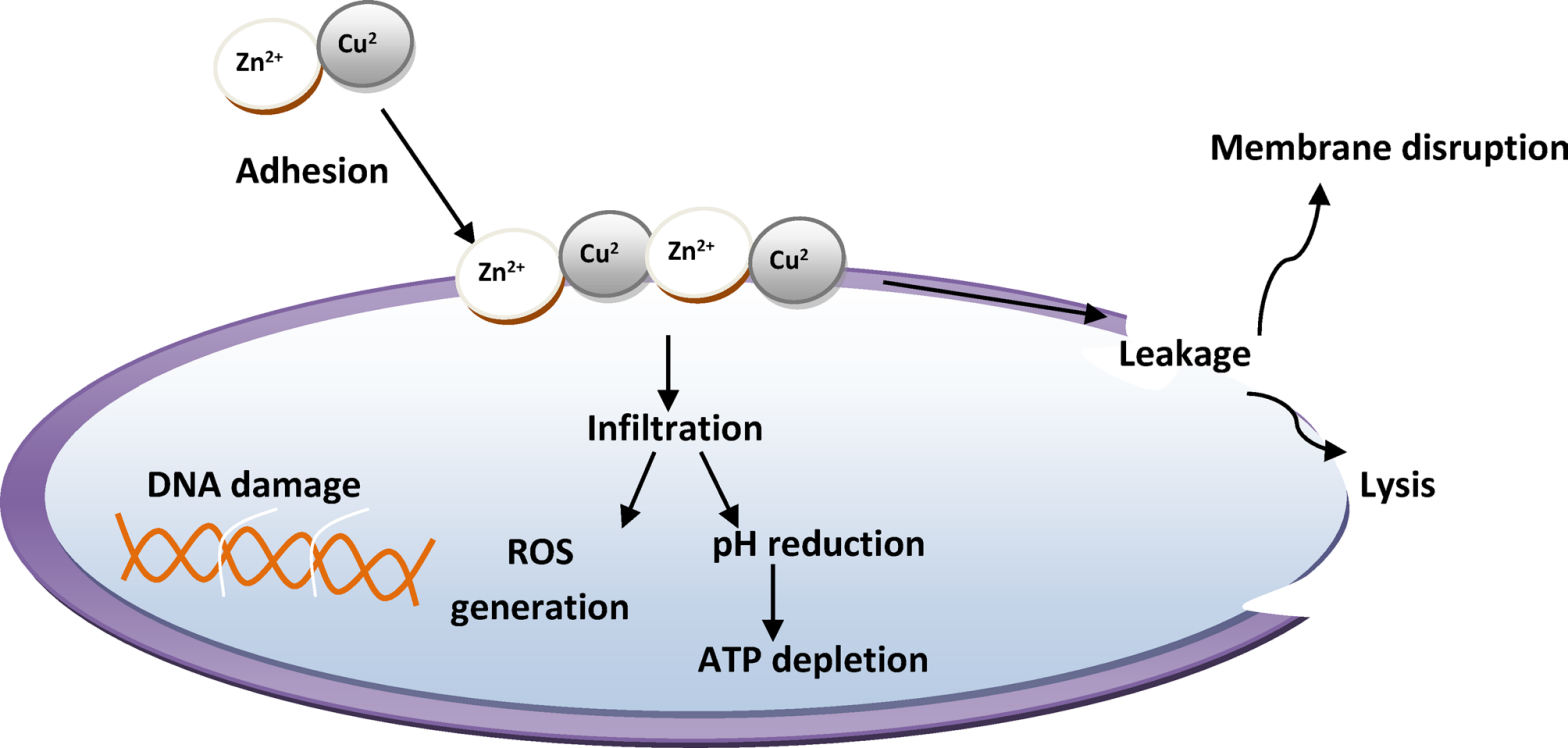
A schematic diagram for antimicrobial mode of action of the prepared nanopigments and nanocomposites

The prepared nanocomposites exhibit antimicrobial activity via several mechanisms, as shown in Fig. 11 as follows:

- By significantly interacting with the soft bases found in proteins, Zn^2+^ and Cu^2+^ can inhibit the action of bacteria and degrade enzyme activity. As a result, the shape of the cell will change, impeding physiologic processes and microbial activity^30^.
- Owing to the small particle sizes and vast surface areas, nanocomposites are more probable to come into contact with microbial cell membranes and cause severe harm^31–33^.
- Moreover, ZnO and CuO are transition metal oxides and semiconductors. ZnO has a broad band gap of 3.3 eV, while the band gap energy of CuO is between 1.2 and 2.6 eV. Electron-hole pairs arise when the radiation’s energy exceeds the band gaps of ZnO and CuO. The conduction band is opened up to electrons. Extremely oxidizing sites that can oxidize water molecules or hydroxide anions and produce strong oxidizing species are developed as a result of the extremely oxidizing nature of the hole that is produced in the valence band. As a result of this reaction, reactive oxygen species (ROS) are produced, with hydroxyl radical ($\:O{H}^{\bullet\:}$), hydroperoxyde radical ($\:H{{O}^{\bullet\:}}_{2}^{-}$) and superoxide radical anion $\:\left({{O}^{\bullet\:}}_{2}^{-}\right)$serving as the routes for bactericidal activity. The good antimicrobial activity of coatings based on nano-ZnO may be due to its wide band gap^34^. The following chemical equations describe how ROS is generated for ZnO.

O_2_ + e^–^➔O_2_^–^

H_2_O + h^+^➔OH^–^ + H^+^.

OH**˙** + OH**˙**➔H_2_O_2_.

O_2_^−^ + H_2_O_2_➔OH^−^ + OH^−^ + O_2_

$$\begin{gathered} OH{\text{ }} + {\text{ }}O_{2} + {\text{ }}organic \to CO_{2} + {\text{ }}H_{2} O \\ \downarrow \\ Cell\;membrane\;of\;microorganism \\ \end{gathered}$$

On the other hand, the results show that *Micrococcus luteus* is more sensitive to the prepared pigments than *Staphylococcus aureus*; this may be because *Staphylococcus aureus* is stronger than *Micrococcus luteus* because there is numerous different resistance genes present in *Staphylococcus aureus*. At least 60 distinct resistance genes have been found in S. aureus so far. This covers genes that provide immunity to the vast majority of antimicrobial agent classes^35^. Also, *Candida albicans* is a strong fungus that can easily resist many antifungals because it is an extremely adaptable microbe and can develop resistance with repeated contact with antifungals. Some of the factors that can lead to antifungal tolerance and resistance include the ability to evade host immune defences, the formation of biofilms, which reduces the accessibility of the antifungal, the selection of spontaneous mutations that boost expression or reduce the susceptibility of the Candida species, altered chromosome abnormalities, over-expression of multidrug efflux pumps, and altered chromosome abnormalities^36^. However, the results reveal that *Candida albicans* is very sensitive to the prepared nanocomposites, so these pigments can be identified as very strong anti-fungal agents.

### Investigation of the physical properties

The color of the pigments is one of their most distinctive characteristics. One of the most important investigations concerning pigments in this regard has to do with figuring out their color coordinates. Table 5 displays the findings for the color coordinates of the coatings containing the synthesized pigments. Regarding coatings containing nano-ZnO, the presented data shows that the amount of color measured in the CIELab system was L*, which is high, and their values are near the value of the standard, which means that whiteness is not affected by adding nano-ZnO. Moreover, by adding ZnO/Si, there has been a very slight decrease in value L* due to the presence of grey silica fume, which slightly affects the degree of whiteness. The value of L* in group I is greater than in group II.

**Table 5:** Color measurements.

Tests	Samples	Commercial one	Group I				Group II				
			Nano-ZnO	ZnO/Si	Nano-CuO	CuO/Si	Nano-ZnO		ZnO/Si	Nano-CuO	CuO/Si
Color	L*	99.4	99.4	99.1	77.27	84.4	99.2		97.74	46.48	78.75
	a*	−0.73	−0.62	−0.60	−0.70	−0.88	−0.58		−0.68	−0.54	0.46
	b*	−0.51	−0.59	−0.75	−0.55	−0.47	0.32-		−0.03	−0.67	−0.23

A tendency towards a decrease in lightness (L*) was observed for the coatings containing nano-CuO. This result was expected due to the black color of nano-CuO, while nano-CuO/si has a grey color, and thus its coatings have higher L* values than those containing nano-CuO due to their higher color lightness.

**Fig. 12:**
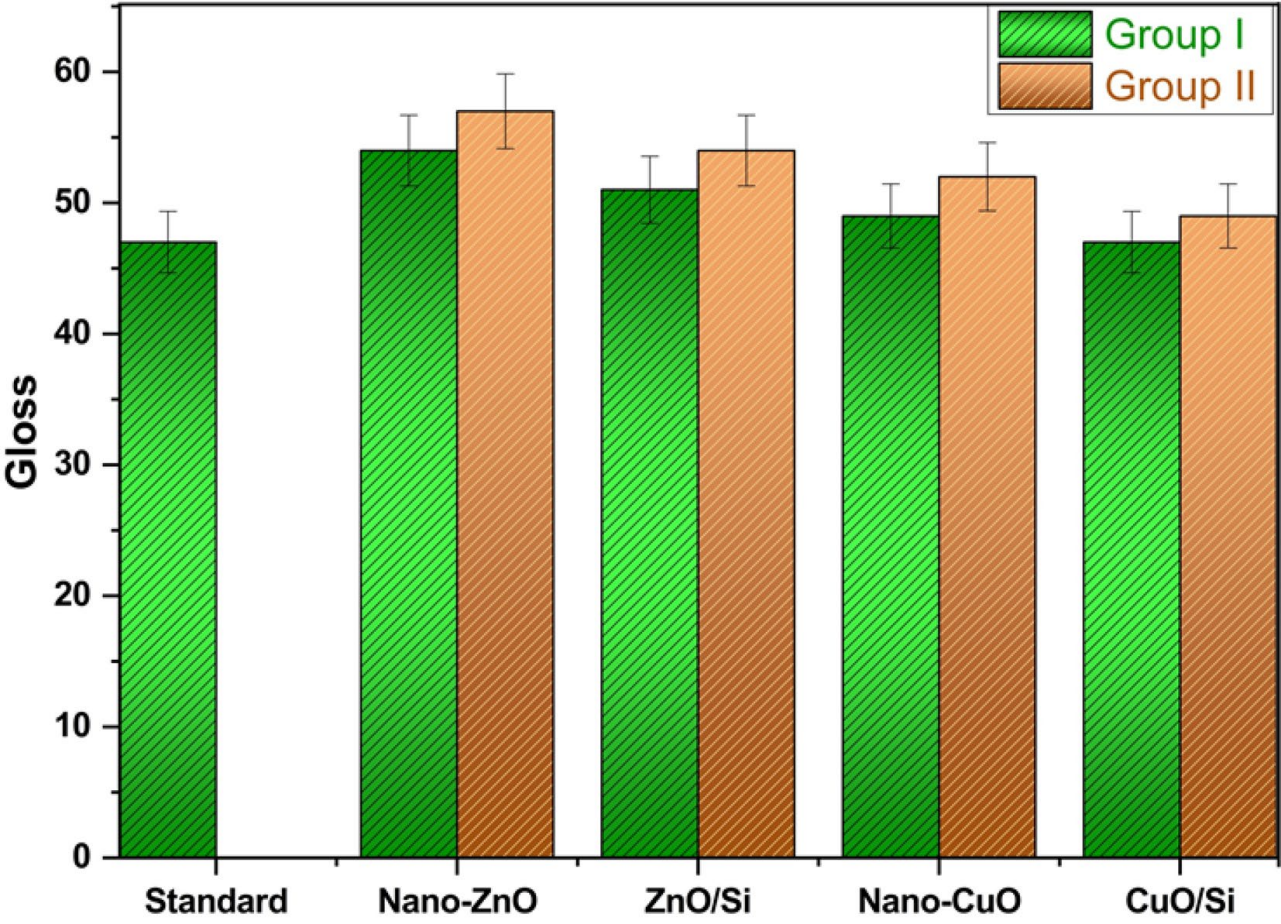
Gloss of the coatings

**Fig. 13:**
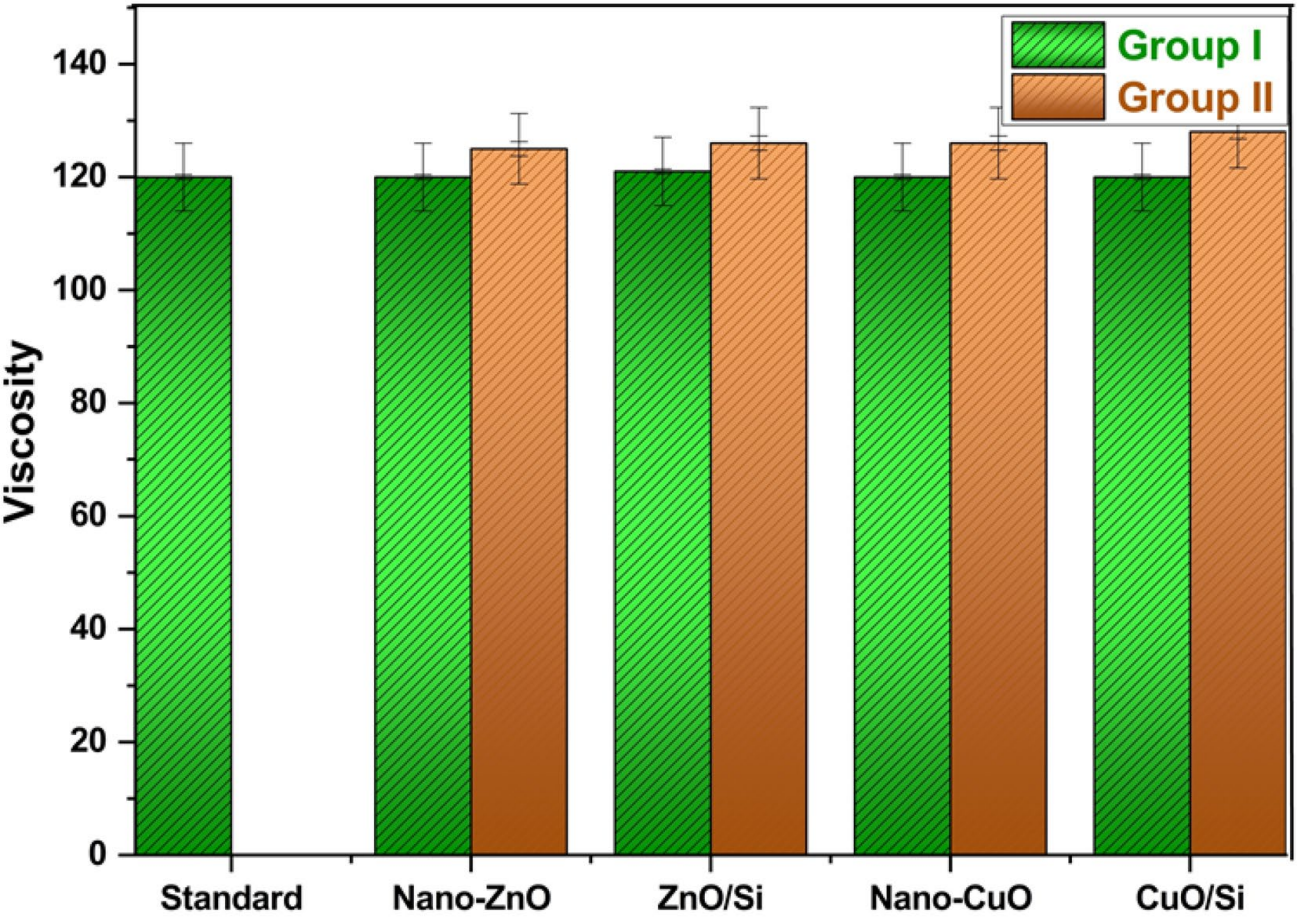
Viscosity of the coatings

Moreover, Fig. 12 containing the gloss results shows that the gloss values of coatings containing the prepared nanocomposites are higher than the commercial one; group II is glossier than group I, which means that gloss is increased by adding the prepared pigments. It is well known that the gloss of the coatings could be identified according to pigment volume concentration (PVC), as when the PVC coefficient is 15–25%, this means that the coatings are high gloss. Here, the prepared coatings and the commercial one have the same PVC coefficient. Thus the difference in the gloss in the present work may be attributed to the presence of Cu²⁺ and Zn²⁺, which enhance the metal’s luster, thus increasing its gloss. Additionally, the results in Fig. 13 show that the viscosity of group I is the same as the standard, and a slight increase has occurred in the viscosity values of group two due to increasing the ratio of the added pigments. Anyway, the viscosity of coatings can be adjusted during the application stage by using the suitable solvent (e.g., here, water is the suitable one).

### Pull-off strength results

The pull-off test was employed in this instance to evaluate the adhesive power of panels with dry and wet coatings. The values for adhesion loss (ψ) were estimated using the equation:

$$\:{\uppsi\:}=({{\upalpha\:}}_{\text{D}}-{{\upalpha\:}}_{\text{W}})/{{\upalpha\:}}_{\text{D}}\text{*}100$$

where α_w_ represents the wet adhesion after immersion in water and α_D_ indicates the dry adhesion.

**Fig. 14:**
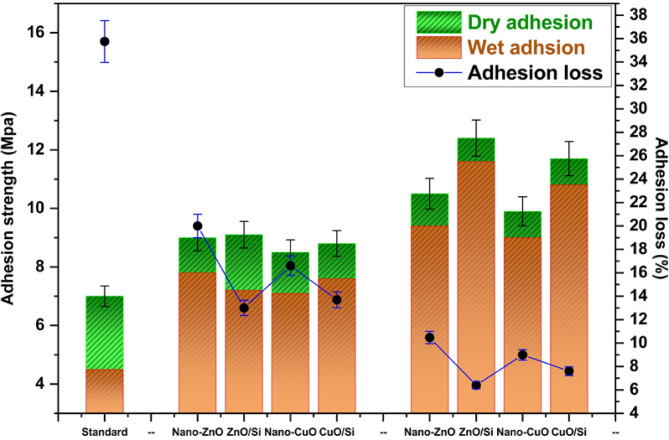
Pull-off test

Figure 14 demonstrates that the panel containing standard coating had the lowest dry and wet adhesion strengths, while its adhesion loss was the highest at about 36%. The buildup of oxide nanoparticles upon the surface of silica fume can result in an enhancement in either wet or dry adhesion by closing the gaps among sphere-shaped silica fume, generating a compact barrier layer, and restricting the spreading of aqueous solution into the interface between coating and substrate^37^. As a result, all coatings incorporating nanocomposites had strong dry and wet adherence and minimal adhesion loss. The panels with nano-ZnO/silica fume and nano-ZnO/silica fume displayed the lowest adhesion loss, at about 9%, followed by ZnO and CuO. The adhesion of coatings containing high ratio of pigments (0.8%) is better than that containing 0.4% of pigments. As the presence of pigments in the polymeric matrix with high ratio could block all voids that can facilitate the penetration of water, so adhesion is improved.

### The mechanical characteristics

**Fig. 15:**
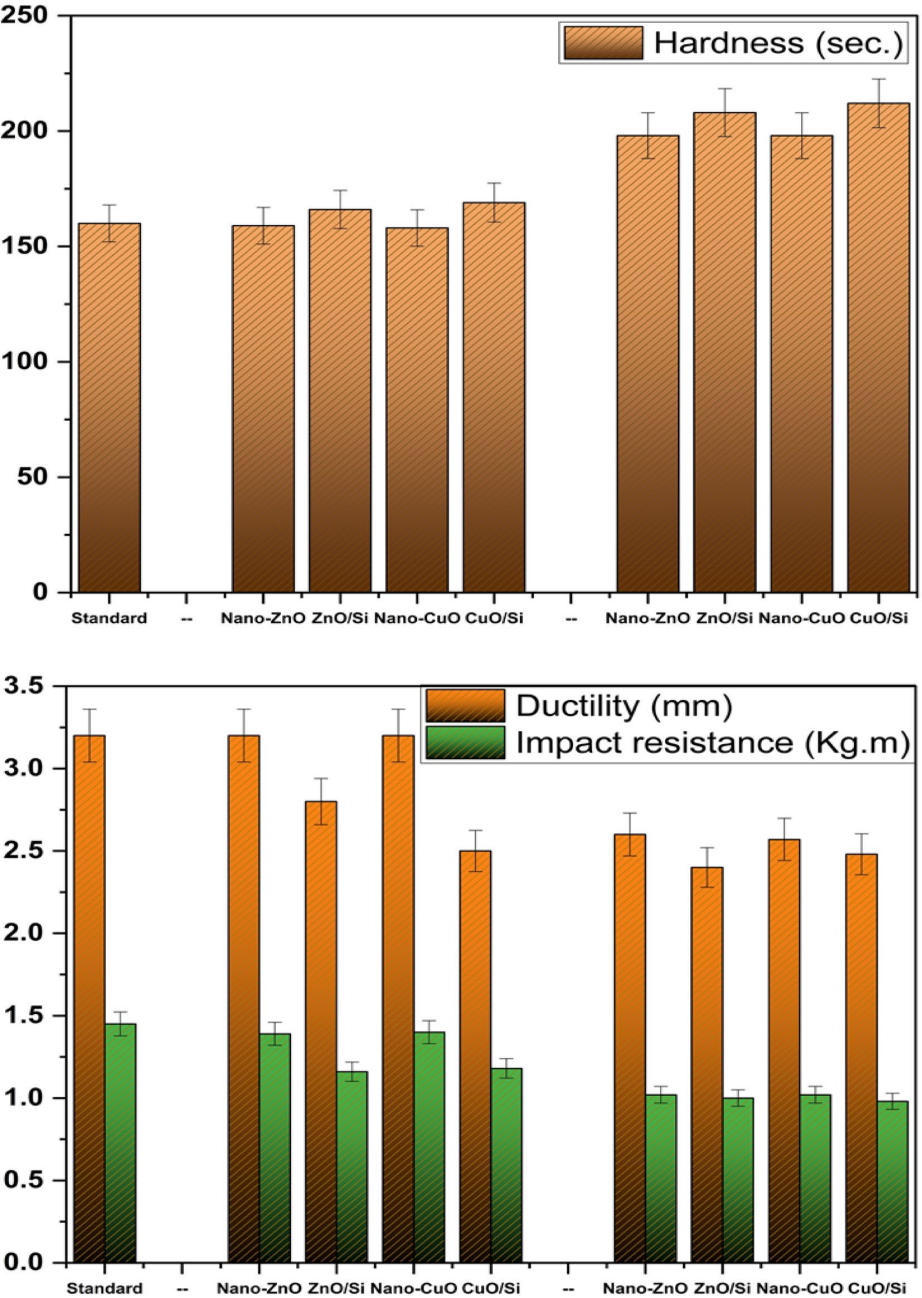
Mechanical properties of the prepared coatings

Figure 15 indicates the mechanical properties of the commercial coatings and the prepared coatings containing nanopigments and nanocomposites. The data declare that the hardness of the coatings containing either nano-ZnO or nano-CuO and the commercial one is almost the same in group I. While the hardness of the coatings containing nano-ZnO/silica fume and nano-CuO/silica fume is slightly higher than that of the commercial one. According to SEM and TEM photos of silica fume, the particles of silica have a spherical structure, and after depositing a thin layer of the nano-oxides on the surface of silica, these nanoparticles can enter and overlap in the voids between spheres of silica and close all pores. Thus, compact particles could be arranged uniformly in the acrylic matrix and form highly built films with good hardness. By increasing the ratio of the nanocomposites in group II, the hardness increased. This also could be attributed to the good distribution of the nanoparticles and nanocomposites in the film can form a highly compact matrix with higher hardness^38,39^.

Additionally, the ability of coatings to undergo elastic or plastic deformation without cracks under external force, which is called ductility, for group I is marginally higher than that for group II. Besides, the ability of the coatings to resist cracking or breaking caused by high mechanical loads and stress levels produced by shrinking or swelling, mechanical abuse, and weathering, which is impact resistance, is decreased by increasing the proportions of the nanocomposites. This could be because the high ratio of the nanocomposites within the acrylic matrix can limit their free mobility, thereby decreasing their flexibility^37^.

The results are summarized in Table 6, which presents the mechanical and physical properties of the coatings. The gloss measurements indicate that the incorporation of nanocomposites enhances gloss levels more significantly than the commercial coating, attributed to the metallic luster of zinc and copper. Besides, viscosity measurements reveal that the values for group I are almost consistent with those in the same group, while group II exhibits the highest viscosity values. Furthermore, the adhesion performance of coatings containing the synthesized nanoparticles surpasses that of the commercial coating.

**Table 6:** The physical and mechanical properties of the coatings.

Samples tests	Commercial one	Group I					Group II				
		Nano-ZnO	ZnO/Si		Nano-CuO	CuO/Si	Nano-ZnO	ZnO/Si		Nano-CuO	CuO/Si
**Gloss**	47	54	51		49	47	57		54	52	49
**Viscosity**	120	120	121		120	120	125		126	126	128
*Mechanical properties*											
Hardness	160	159		166	158	169	198		208	198	212
Ductility	3.2	3.2		2.8	3.2	2.5	2.6		2.4	2.5	2.4
Impact resistance	1.45	1.39		1.16	1.4	1.18	1.02		1	1.02	0.98
*Adhesion*											
Dry adhesion	7	9.1		9	8.5	8.8	10.5		12.4	9.9	11.7
Wet adhesion	4.5	7.2		7.8	7.1	7.6	9.4		11.6	9	10.8
Adhesion loss	35.75	20.8		13.3	16.4	13.6	10.4		6.4	9	7.6

Regarding the mechanical properties, the hardness of the coatings in group I is comparable to that of the commercial one. However, an increase in the proportion of nanoparticles correlates with an enhancement in hardness. Conversely, ductility and impact resistance values decrease with higher nanoparticle ratios.

### Advantage of the new coatings containing the prepared nanocomposites in comparable to the commercial coating

- In these coatings, the antibacterial agent, which is imported and very expensive, was replaced with local antimicrobial pigments that are prepared locally. This will help save hard currency for the country.
- The prepared nanocomposites are synthesized using a simple and inexpensive method, besides both nano-ZnO/silica fume and nano-CuO/silica fume are based on 90% of industrial waste (e.g., silica fume). This means that the cost of the final product will be low, and the results showed that some of the developed coatings have succeeded in offering higher antimicrobial activity than the commercial ones.
- The prepared nanocomposites contain low concentrations of heavy metals (Zn or Cu), so they are safe and eco-friendly.

## Conclusions

In the framework of this study, novel antimicrobial nanocomposites were created using cost-effective industrial waste by depositing a thin layer of nano-ZnO or nano-CuO on silica fume, which makes up 85% of the composite. After synthesizing and characterizing the nanocomposites, they were incorporated into acrylic waterborne coatings at 0.4% and 0.8% weight ratios. The antimicrobial efficacy was tested against *Staphylococcus aureus* and *Micrococcus luteus* (bacterial strains) and *Candida albicans* (a pathogenic fungus) using the disc diffusion and shake flask methods. Results showed that the commercial coating produced inhibition zones of 16 to 21 mm, while the 0.8% nano-ZnO/silica fume disc exhibited the highest antimicrobial activity, with zones ranging from 17 to 26.6 mm. Additionally, coatings with nano-ZnO outperformed those with nano-CuO. In terms of mechanical properties, the hardness of coatings with either nano-ZnO or nano-CuO was similar to that of the commercial coatings in group I. However, coatings with nano-ZnO/silica fume and nano-CuO/silica fume showed slightly higher hardness. In group II, higher ratios of nano-ZnO, nano-CuO, and their silica fume composites significantly enhanced hardness compared to the commercial coatings, attributed to a more compact film formation. Furthermore, coatings with a higher pigment ratio (0.8%) demonstrated better adhesion than those with a lower ratio (0.4%). The overall results indicate that the discs containing 0.8% nano-ZnO/silica fume demonstrated superior antimicrobial activity compared to the commercial coatings. This pigment comprises 15% nano-ZnO and 58% industrial waste (silica fume), establishing it as a cost-effective antimicrobial agent. Thus, the formulation not only enhances antimicrobial efficacy but also promotes sustainability by utilizing industrial byproducts.

## Data Availability

The datasets used and/or analyzed during the current study available from the corresponding author on reasonable request.
